# Parental clinical manifestation association with newborn immune senescence and telomere biology in Pakistan

**DOI:** 10.1186/s13104-025-07498-4

**Published:** 2025-10-30

**Authors:** Sadia Farrukh, Saeeda Baig

**Affiliations:** 1 Department of Biological and Biomedical Sciences, Aga Khan University, Stadium Road, P.O. Box 3500, Karachi, 74800 Pakistan; 2 Department of Biochemistry, Ziauddin University and Fazaia Ruth Pfau Medical College, Karachi, Pakistan

**Keywords:** Telomere, Telomere length, Telomerase, TERC, TERT, Immune markers, Newborn, CD57, KLRG1

## Abstract

**Objective:**

This study investigates the association of parental clinical manifestations with newborn telomere biology and immune senescence markers, utilising 204 parent–newborn triads in Karachi, Pakistan. The demographic data collection was followed by quantification of telomere length (TL) using quantitative PCR, while Sanger sequencing was performed to analyse variants in telomerase genes [Telomere Reverse Component (TERC) and Telomerase Reverse Transcriptase (TERT)]. Moreover, flow cytometry was used to analyse immune senescence markers CD57 and Killer cell lectin-like receptor G1 (KLRG1).

**Results:**

The study revealed that immune senescence markers (CD57⁺KLRG1⁺) (3.5 ± 5.49, 3.1 ± 1.27) were significantly overexpressed in newborns from the diseased parent (diabetes, hypertension) (*p* = 0.04), and particularly KLRG1^+^ expression was positively correlated with both maternal and paternal TLs (mother: r = 0.395; *p* = 0.003**,** father: r = 0.32; *p* = 0.014). Parents with diseases (chronic/acute) exhibited shorter TLs (mother: 1.54 ± 1.37, 0.98 ± 0.81; father: 1.32 ± 1.1, 1.18 ± 0.94) compared to their newborns (2.32 ± 1.43, 2.2 ± 1.47) (*p* = 0.048). Furthermore, genotypic analysis revealed a predominance of the C/C genotype of TERT (rs2736100), which showed significant associations with diabetes [10 (50%)] and hypertension [9 (56%)] (*p* = 0.001). The Newborns of parents with clinical manifestations of diabetes exhibited upregulation of KLRG1 markers and a correlation with shorter telomere length.

**Supplementary Information:**

The online version contains supplementary material available at 10.1186/s13104-025-07498-4.

## Introduction

Immunity ensures tissue homeostasis, but with age, its decline leads to immune senescence [1]. Telomere shortening driven by genetic and epigenetic factors is a key marker of this process and aging [2, 3] and is maintained by an enzyme called telomerase. The Telomerase RNA component (TERC) is widely expressed in cells, while telomerase reverse transcriptase (TERT), its protein component, has limited expression in somatic tissues but is active in germ cells, stem cells, and lymphocytes, making TERT a key regulator of telomerase activity. Like DNA polymerase, telomerase extends chromosome ends by adding nucleotide repeats [4]. Research has shown that telomere length (TL) shortening or TERT mutations can be inherited and are linked to diseases like dyskeratosis congenita, aplastic anemia, and pulmonary fibrosis [5]. However, limited data exist on how environmental factors or diseases like diabetes, hypertension, and COVID-19 affect leukocyte telomere length (LTL) and its transmission to the next generation.

Telomere shortening activates cell cycle inhibitors (p53, p21, p16) and increases senescent T cells by suppressing cyclins and Cdks [1]. Killer cell lectin-like receptor G1 (KLRG1) and Cluster of Differentiation 57 (CD57) are key markers of immune senescence, with KLRG1 primarily expressed on natural killer (NK) cells, senescent T cells, and CD57 found on both CD4⁺ and CD8⁺ T cells [6, 7]. With aging, highly differentiated T cells accumulate, showing impaired proliferation, Akt signaling pathway, and telomere maintenance by telomerase, reflecting near-replicative senescence. Therefore, KLRG1 is a potential marker that reflects immune senescence and is involved in cellular aging [8–10]. However, premature T-cell immune senescence, is also marked by the accumulation of CD57⁺ CD8⁺ T cells, has been associated with both immunodeficiency and autoimmunity. These cells typically lack the chemokine receptors CCR7 and CD45RO, show reduced expression of the costimulatory molecules CD27 and CD28, and exhibit upregulation of CD45RA [11, 12].

In senescence cells, telomerase expression and activity are reduced, which ultimately promotes DNA damage by phosphorylation of the p38, ERK, JNK, and STAT signalling pathways and causes telomere shortening by a decrease in expression and activity of telomerase, which can initiate inflammaging [12, 13]. The functional failure of exhausted T cells, like in Type 2 diabetes mellitus (T2DM), that fail to uptake glucose can be restored through immune checkpoint blockades, but energy imbalance may also be the root of their functional impairment, leading to immune senescence [14–16].

In our Previous research [17], it was found that immune senescence is more pronounced in parents than in newborns. Hence, it's crucial to investigate parental risk factors and their impact on newborns, particularly how parental clinical conditions influence immune senescence markers, TL, and telomerase-related genes (TERC and TERT). Therefore, this study was designed to investigate the impact of parental clinical manifestations on telomere biology and immune senescence markers in newborns.

## Materials and methods

A total of 612 participants (204 mother-father-newborn triads) were enrolled after ethics approval (ERC Ref No. 3950721SFBC). Samples were collected from September 2021 to June 2022 using convenience sampling, with informed consent from different hospital centres of Ziauddin University, Karachi, Pakistan. For demographics, age and socioeconomic status (SES) assessed using participants’ income (based on the dollar exchange rate as of 1 May 2022), were collected through a detailed questionnaire (see Supplementary File). Eligible participants included females aged 18–35 and males aged 18–45 with different diseases, excluding those with known cancers. Participants were categorised into chronic (diabetes, hypertension, anaemia) and acute (COVID-19) disease groups. Blood samples (5 ml) from each parent and umbilical venous cord blood (5 ml) from newborns (gestational age > 37) were collected with the help of a syringe in EDTA tubes, and then stored at 4 °C. The DNA was extracted using the Qiagen DNA Blood Mini Kit (catalog # 51,306, Germany) and its concentrations were measured using a spectrophotometer (Multiskan Sky, Thermo Fisher Scientific, USA) at 260 and 280 nm, The ratio of 1.8 (260/280) was considered for pure DNA and stored at − 80 °C for further analysis.

### Leukocyte telomere length quantification by qPCR

Leukocyte telomere length (TL) was measured by qPCR using the multiplex method [17]. All qPCR reactions included the reference DNA (n = 4)(pooled blood from two healthy males/females) as a standard in all runs of qPCR. The standard curve was created using the 5 dilutions with a range of 150–1.85 ng of reference DNA. The experimental mother, father, and cord DNA were then measured using qPCR. The qPCR reaction procedure was done according to our published work [17].

### Sanger sequencing for TERC and TERT gene polymorphism

Following qPCR, further chronic/acute diseased (n = 53) and healthy (n = 10) participants were selected and subjected to Sanger sequencing for polymorphism detection. Variants of the TERC (rs10936599) and TERT (rs2736100) genes were chosen based on their association with telomere length and a minor allele frequency (MAF) > 5% in the Pakistani population, as identified from the 1000 Genomes database (https://www.ncbi.nlm.nih.gov/variation/tools/1000genomes/). The gene loci for TERC (617 bp) and TERT (544 bp) were amplified using conventional PCR. The primer sequences used are TERC forward: 5′-CAGGTTTTGCTGTGAACTCGG-3′, reverse: 5′-GACTACTGACTAGTCTCAGG-3′ and TERT forward: 5′-AAGCGTCCTCATCCTTTGT-3′, reverse: 5′-TCTCAGGCATCTTGACACCC-3′ (Synbio Tech, USA). Gene loci were amplified using specific primers and thermal cycling conditions, followed by established protocols [17].

### Flow cytometry for immune senescence detection

A further flow cytometry technique was used for immune senescence detection. Participants were selected and grouped based on chronic and acute diseases (n = 53). The healthy (n = 10) group was considered as a control. Blood samples were collected in EDTA tubes within 24–48 h and used to isolate Peripheral Blood Mononuclear Cells (PBMCs), which were cryopreserved. Cells were initially stored at − 20 °C, then transferred to –80 °C for long-term preservation. For analysis, PBMCs were revived in culture media with FBS, suspended in PBS, and stained with 1 μL of monoclonal antibodies: FITC-CD57, PerCP-CD45, and PE-KLRG1 (Thermo Fisher). After a 30-min incubation at 4 °C in the dark, flow cytometry was performed using FACS Calibur (BD Biosciences). Lymphocytes were gated using Forward Scatter/Side Scatter (FSC/SSC), to assess lymphocyte size and complexity then CD45 + cells were analyzed for CD57/KLRG1 markers. Data were visualised via dot plots and histograms using FACS DIVA software, distinguishing CD57⁺, KLRG1⁺, double-positive, and double-negative cells.

### Statistical analysis

The Statistical Package for Social Sciences (SPSS)(version 27) and GraphPad Prism Software (version 10.1.2) were used to analyze the data. The qualitative variables were calculated as frequencies and percentages, whereas the quantitative data were calculated as means and standard deviation (SD). The Mann–Whitney U test was used to compare the effects of chronic and acute diseases in both newborns and parents. The correlation between parents-newborn TL (T/S ratio) and immune markers was done by Pearson correlation. The mean difference between diseases, TL, TERC & TERT genes and immune senescence markers was done by ANOVA and chi-square test for results analysis. The statistical significance was defined as *p* < 0.05.

## Results

A total of 612 participants were recruited for the study; the mean age of the mothers was 27 ± 3.12 years, while the mean age of the fathers was 34 ± 3.36 years. Parents aged less than 25 had longer TL (Mother (M): 1.54 ± 1.18, Father (F): 1.73 ± 1.14) compared to those above 35 years (p = 0.04). In comparison, newborns (N) to younger parents had smaller TL (1.85 ± 1.40) than older parents (2.38 ± 1.62) (*p* = 0.04). Demographic analysis revealed significantly shorter telomere lengths (TL) in low socioeconomic status (SES) (1.95 ± 1.36, *p* = 0.000). Newborns of parents with pre-secondary education showed significantly shorter TL (1.92 ± 1.41; *p* = 0.007). Among white-collar jobs, newborns of homemaker mothers (2.04 ± 1.48) and labourer father (2.07 ± 1.32) had the shorter TLs (*p* = 0.000). Furthermore, healthy parents showed longer TLs than those with chronic (M: 1.54 ± 1.37, F: 1.32 ± 1.10) or acute diseases (M: 0.98 ± 0.81, F: 1.18 ± 0.94), while their newborns had significantly longer TLs (2.32 ± 1.43, 2.2 ± 1.47; *p* = 0.048) (Table 1).

**Table 1:** Mean difference between demographic and T/S ratio in parents and their newborns

Variables		Mother n = 204		Father n = 204		Newborn n = 204	*p*-value
		*n* (%)	TL (T/S Ratio) (Mean ± SD)	*n* (%)	TL (T/S Ratio) (Mean ± SD)	TL (T/S Ratio) (Mean ± SD)	
Age (yrs.)	< 25	56 (27)	1.54 ± 1.18	11 (5)	1.73 ± 1.14	1.85 ± 1.40	0.040*
	25–35	143 (70)	1.52 ± 1.30	114 (60)	1.57 ± 1.12	2.31 ± 1.46	
	> 35	5 (3)	1.49 ± 1.23	79 (39)	1.38 ± 1.01	2.38 ± 1.62	
Socioeconomic status	Low	102 (50)	1.5 ± 1.14	102 (50)	1.41 ± 1.08	1.95 ± 1.36	0.000*
	High	102 (50)	1.93 ± 1.37	102 (50)	1.70 ± 1.12	2.05 ± 2.21	
Occupation	Blue collar						
	Homemakers	164 (80)	1.57 ± 1.26	N/A	N/A	2.04 ± 1.48	0.000*
	Laborer	N/A	N/A	28 (14)	1.42 ± 1.2	2.07 ± 1.32	
	Shopkeeper	N/A	N/A	7 (3)	1.57 ± 1.02	2.16 ± 1.09	
	Doctor	8 (4)	1.69 ± 1.20	11 (5)	1.64 ± 1.23	2.19 ± 1.35	
	White collar						
	Business	N/A	N/A	43 (21)	1.67 ± 1.15	2.21 ± 1.10	0.000*
	Private job	19 (9)	1.67 ± 1.25	115 (56)	1.37 ± 1.20	2.35 ± 1.20	
	Teacher	13 (6)	1.62 ± 1.24	N/A	N/A	2.57 ± 1.27	
Education	Pre-secondary and secondary	121 (59)	1.55 ± 1.20	116 (57)	1.38 ± 1.08	1.92 ± 1.41	0.007*
	Post-secondary	83 (41)	1.88 ± 1.35	88 (43)	1.69 ± 1.11	2.32 ± 1.44	
Health status	Healthy	46 (22)	1.89 ± 1.42	121 (60)	1.66 ± 1.18	2.34 ± 1.20	0.048*
	Chronic diseases	138 (68)	1.54 ± 1.37	68 (33)	1.32 ± 1.1	2.32 ± 1.43	
	Acute diseases	20 (10)	0.98 ± 0.81	15 (7)	1.18 ± 0.94	2.2 ± 1.47	

*p value: significant N/A: Not Available

Chronic Diseases: Diabetes, Hypertension, Anemia Acute diseases: COVID-19

**Fig. 1:**
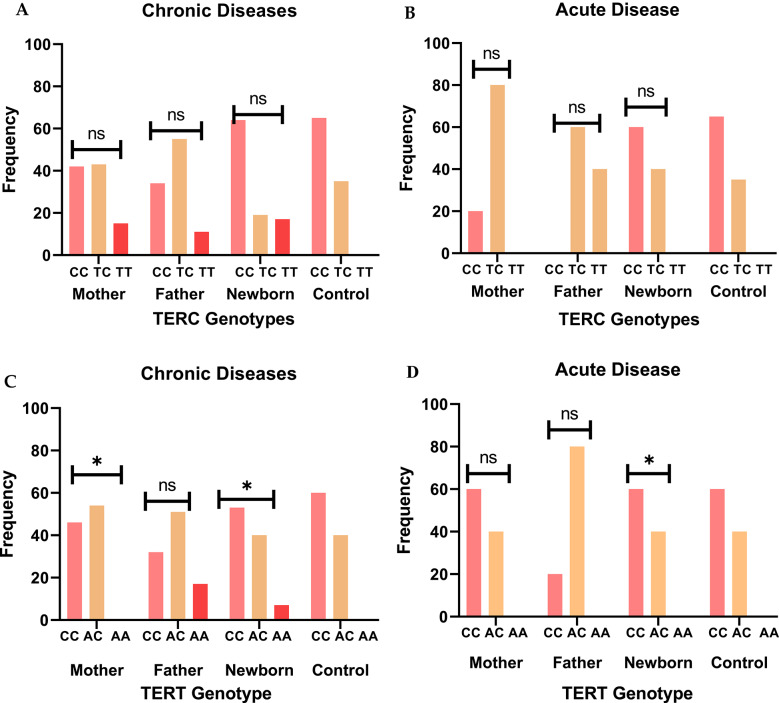
TERC and TERT genotype distribution among chronic and acute diseases in parents and newborns. The CC genotype was dominant in newborns with chronic (**A**) and acute diseases (**B**), whereas the TT genotype was seen only in chronic diseases. The TERT genotype CC was dominant in newborns with chronic (53%) and acute disease (60%) in newborns (**C** and **D**) with significant results (*p* = 0.00). ns: non-significant, * *p*-value: significant

In Fig. 1A, B, TERC genotypes of telomerase enzyme were explored, and it was seen that in the disease group, heterozygous and homozygous genotypes CC, TC and TT were found. The overall effect of chronic diseases in newborns showed the more CC genotype (64%) compared to both parents having TC (M:42%, F:55%) (*p* = 0.89). Whereas, in acute diseases, only the genotype CC (60%) was seen in newborns and the TC in parents (M:80%, F:60%) (*p* = 0.33). However, the results were not statistically significant for the TERC gene analysis.

Figure 1C, D highlight the overall impact of chronic and acute disease prevalence across different TERT genotypes in mothers, fathers, and newborns. TERT gene polymorphism also revealed three genotypes: CC, AC, and AA. Genotype AA was not found in mothers. Moreover, among chronic diseases, the genotype AC (M:54%, F:51%) was found in parents, whereas CC (53%) was in newborns (*p* = 0.01).

**Fig. 2:**
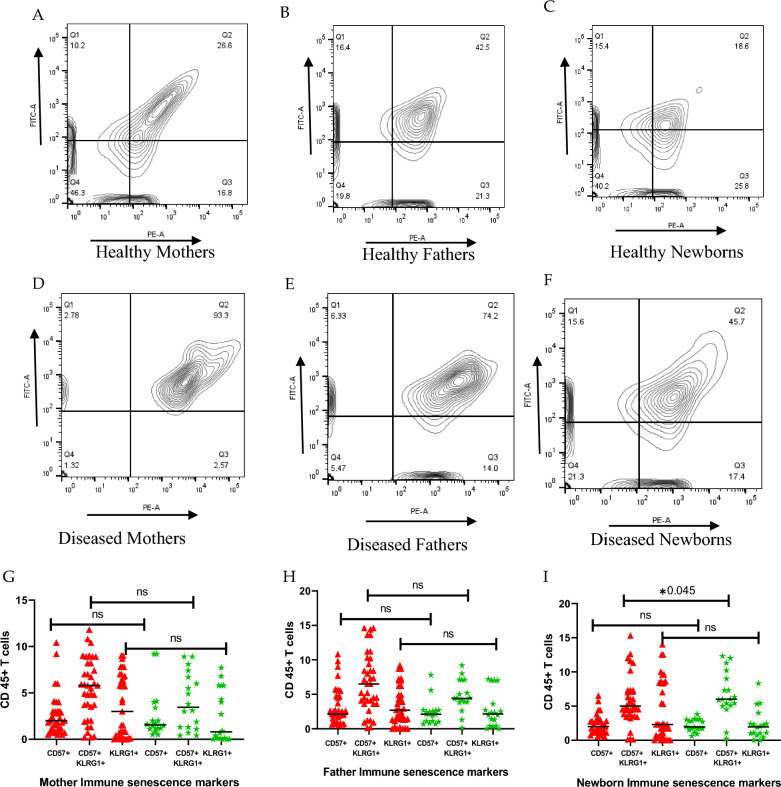
Immune senescence markers (CD57, KLRG1) in Parents and newborns. Healthy Mothers(M), Fathers(F) and Newborns(N).** A**,** B**,** C** had decreased expression of CD57 + KLRG1 + (M:26.6%, F:42.5%, N:18.6%) compared to diseased (**D**,** E**,** F**) which showed upregulation of immune markers (M:93.3%, F:74.2%, N:45.7%). Non-significant results were observed when the disease and healthy groups of mothers and fathers were compared (**G**,** H**). Significant expression of CD57 + KLRG1 + was seen in newborns (*p* = 0.045) (**I**). Red tiles: Diseased; Green tiles: Healthy; ns: non-significant;*: significant)

Analysis of the TERC and TERT genes revealed a high frequency of the CC genotype in newborns of parents with chronic diseases, particularly diabetes [ TERC: 16 (79%)] (*p* = 0.079), TERT: 10(50%) (*p* = 0.00)]. Additionally, the AA genotype [3(15%)] was found in newborns but was absent in mothers. In acute diseases, statistical analysis revealed a significant association between the TERT genotype and disease susceptibility in newborns (*p* = 0.01), suggesting a potential genetic influence (Supplementary Tables and Figs. 1, 2).

Flow cytometry analysis was performed to assess immunosenescence in parents and their newborns. In cases of parent-newborn diseases, the mean expression of immune senescence markers (CD57⁺KLRG1⁺) on senescent T cells and natural killer cells was elevated (M:93.3%, F:74.2%, N:45.7%), while a decreased expression of markers was observed in healthy parent-newborn pairs (M:26.6%, F:42.5%, N:18.6%) (Fig. 2). Significant results were only seen in newborns with decreased expression compared to parents (*p* = 0.045) (Fig. 2I).

In Table 2, the immunosenescence analysis revealed that healthy parents and their newborns had longer TL (M:1.89 ± 1.42, *p* = 0.89; F: 1.66 ± 1.18, *p* = 0.11; N: 2.32 ± 1.43, *p* = 0.67) compared to diseased parents. Moreover, when further analysing the diseases, newborns of parents with chronic conditions, including diabetes and hypertension, had shorter telomere lengths (2.02 ± 1.36, 1.48 ± 1.17) and increased expression of immune markers (3.5 ± 5.49, 3.1 ± 1.27) with the difference being statistically significant (*p* = 0.04) (Table 2).

**Table 2:** Comparison of parents ' newborn immune senescence markers in different diseases

Parameters (Mean ± SD)	Mother n = 204				Father n = 204				Newborn n = 204			
	Telomere length TL (T/S Ratio)	CD57 +	CD57 + KLRG1 +	KLRG 1 +	Telomere length (T/S ratio)	CD57 +	CD57 + KLRG 1 +	KLRG1 +	Telomer e length (T/S ratio)	CD57 +	CD57 + KLRG 1 +	KLRG 1 +
Healthy n = 10	1.89 ± 1.42	2.5 ± 2.58	4.16 ± 2.97	3.25 ± 4.26	1.66 ± 1.18	2.36 ± 1.85	4.23 ± 4 29	3.61 ± 4.19	2.32 ± 1.43	2.20 ± 0.87	2.45 ± 4.34	1.74 ± 4.23
Diabetes n = 20	1.54 ± 1.37	2.09 ± 2.43	6.63 ± 3.57	3.40 ± 3.43	1.32 ± 1.1	3.2 ± 2.52	6.82 ± 4.76	4.78 ± 3.3	2.02 ± 1.36	2.41 ± 1.28	3.5 ± 5.49	3.44 ± 3.96
Hypertension n = 16	1.36 ± 1.02	5.0 ± 5.9	5.4 ± 4.94	3.31 ± 3.8	1.41 ± 0.91	2.57 ± 0.39	5.83 ± 3.23	4.07 ± 6.95	1.48 ± 1.17	3.4 ± 0.56	3.1 ± 1.27	1.2 ± 9.61
Diabetes & Hypertension n = 5	1.29 ± 1.02	2.56 ± 2.61	4.6 ± 5.02	3.27 ± 3.79	1.46 ± 1.16	2.48 ± 2.15	6.98 ± 4.68	8.95 ± 9.63	2.28 ± 1.54	2.80 ± 0.34	2.32 ± 2.51	1.15 ± 1.32
A*n*emia n = 7	1.68 ± 1.26	2.75 ± 2.70	5.3 ± 3.07	2.2 ± 3.67	1.26 ± 1.09	2.2 ± 1.25	5.28 ± 4.89	4.76 ± 2.3	2.02 ± 1.03	2.91 ± 2.08	3.15 ± 3.49	2.04 ± 2.61
COVID-19 n = 5	1.08 ± 0.81	3.32 ± 1.41	2.12 ± 1.27	3.92 ± 2.72	1.18 ± 0.9	5.43 ± 4.09	5.16 ± 5.05	7.53 ± 3.68	2.2 ± 1.47	2.35 ± 0.3	2.1 ± 2.82	2.5 ± 3.96
P value	0.89	0.91	0.02*	0.008*	0.11	0.87	0.60	0.14	0.67	0.21	0.04*	0.18

*p value: significant

**Fig. 3:**
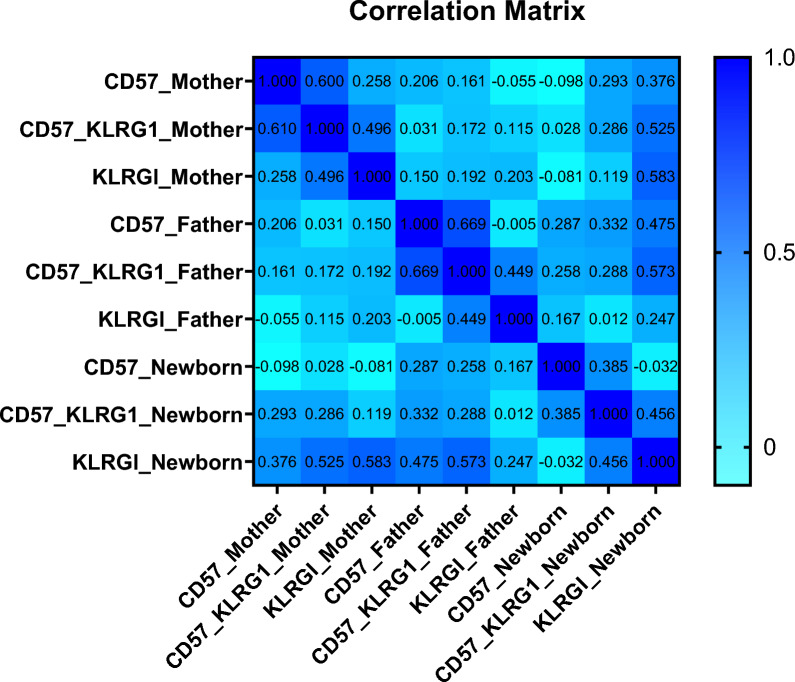
Correlation matrix among the immune senescence markers of parents and newborns. Correlation analysis showed a positive association between parental and newborn immune senescence markers (CD57 KLRG1), highlighting a positive relation of both maternal (r = 0.286; *p* = 0.035) and paternal (r = 0.288; *p* = 0.033) markers

Correlation analysis showed a positive association between parental and newborn immune senescence markers (CD57 KLRG1), highlighting a positive relation of both maternal (r = 0.286; *p* = 0.035) and paternal (r = 0.288; *p* = 0.033) markers. Moreover, a more positive correlation was found between maternal and newborn KLRG1 (r = 0.583; *p* = 0.000), indicating greater maternal influence on newborn immune aging (Fig. 3, Supplementary Table 3). Additionally, parental telomere length (T/S) was positively correlated with newborn KLRG1⁺ levels (M: r = 0.329; *p* = 0.14, F: r = 0.395; *p* = 0.003) while newborn telomere length negatively correlated with its CD57 expression (r = − 0.269; *p* = 0.047) (Table 3, supplementary Fig. 3).

**Table 3:** Correlation analysis among immune senescence markers CD57 and KLRG1 of newborns and T/S ratio in diseased parents

Immune markers		CD57 KLRGI-Newborn	KLRGI-Newborn	CD57-Newborn	T/S-Mother	T/S-Father	T/S-Newborn
CD57 KLRGI-Newborn	r	1.000	0.456	0.385	0.123	0.177	0.114
	*p*-value		0.000	0.004	0.369	0.200	0.409
KLRGI-Newborn	r	0.456	1.000	− 0.032	0.329	0.395	0.150
	*p*-value	0.000		0.818	0.014	0.003	0.275
CD57-Newborn	r	0.385	− 0.032	1.000	− 0.059	0.110	− 0.269
	*p*-value	0.004	0.818		0.667	0.430	0.047
T/S-Mother	r	0.123	0.329	− 0.059	1.000	0.331	0.425
	*p*-value	0.369	0.014*	0.667		0.000	0.000
T/S-Father	r	0.177	0.395	0.110	0.331	1.000	0.355
	*p*-value	0.200	0.003*	0.430	0.000		0.000
T/S-Newborn	r	0.114	0.150	− 0.269	0.425	0.355	1.000
	*p*-value	0.409	0.275	0.047*	0.000*	0.000*	

*p value: significant

It was found that mothers' and fathers' TL (T/S) are positively correlated with newborn immune senescence marker (KLRG1 +)(Mother: r = 0.395; *p* = 0.003), (Father: r = 0.32; *p* = 0.014)

## Discussion

### Immune senescence markers affected by parental diseases

Clinical manifestations, including diabetes and hypertension in parents, upregulate immune senescence markers in utero were examined for the first time in the Pakistani population and have not been reported before in the literature. The telomere length (TL) of lymphocyte subsets (senescent T-cells, NK cells) between parents and neonates, particularly in different diseases, was also observed for the first time. It was discovered that parents with the diseases had newborns with upregulated immune markers (M:93.3%, F:74.2%, N:45.7%) (Fig. 2) with positive correlation (M: r = 0.286; *p* = 0.035**;** F: r = 0.288; *p* = 0.033)(Fig. 3), which was consistent with a study that highlighted adults with more expression of markers (r = 0.48, *p* = 0.002) than young children (4–8.5 months) [18].

Fathers with diabetes and hypertension showed elevated immune senescence markers and reduced telomere length compared to healthy fathers (Table 2). Similar trends were observed in tuberculosis patients, though no effect was seen in their infants [19]. This upregulation of immunological senescence markers in fathers may provide important clues about the immune system's participation in the etiology of societal health inequities, occupational hazards, chronic stress and biological wear-and-tear in fathers and their transmission to their infants.

Mothers with diabetes and their newborns exhibited elevated levels of immune senescence markers (CD57⁺KLRG1⁺)(M: 6.63 ± 3.57, N: 3.5 ± 5.49), *p* = 0.02) (Table 2). This finding is consistent with previous studies on patients with type 2 diabetes mellitus (T2DM), with systemic inflammation [16]. Maternal metabolic conditions like diabetes may affect fetal immune development and contribute to immune-related disease transmission. In contrast, COVID-19 mothers and newborns (M: 2.12 ± 1.27, N: 2.1 2.82) (Fig. 2) showed downregulated immune markers, aligning with studies linking low NK cell levels or NK cell exhaustion due to prolonged antigen exposure and disease severity [20, 21].

A positive correlation (M: r = 0.395; *p* = 0.003, F: r = 0.32; *p* = 0.014) (Table 3) between newborn KLRG1 and their parents' TL was also seen in this study, which emphasizes the fact that expression of immunological senescence markers in newborns with TL alterations might be employed significantly as a marker of biological aging of T cells [18, 22, 23] or a reduction in the body's adaptive immunological response [24].

### Telomere length modification under the influence of parental diseases

Looking deep down into telomere alterations, the parental diseases like diabetes nd hypertension showed a significant association not only with their own (parents') TL but also with the newborns. Shorter telomere length (TL) was observed in newborns of parents with a history of COVID-19, indicating a potential association between prior COVID-19 infection and telomere attrition. This was supported by a group of researchers from Spain, who found an association of shorter telomeres with increased severity of COVID-19 infection when measured among patients between the ages of 29 and 85 years old [25]. Moreover, diabetic mothers had shorter telomeres (1.54 ± 1.37) (Table 2), consistent with a study showing that individuals with latent autoimmune diabetes of adulthood (LADA) had shorter telomeres, particularly when compared to patients treated with metformin and insulin[26]. Oxidative stress is one of the known factors to cause telomere shortening, contributing to cardiovascular diseases like hypertension [5]. In this study, hypertensive mothers had newborns with significantly shorter telomeres, suggesting an intergenerational effect. Conversely, longer telomeres are linked to increased cellular lifespan but may pose a germline risk for cell immortality leading to cancer development and progression [27].

### TERT and TERC polymorphism and disease progression

This study is the first to report TERC (rs10936599) and TERT (rs2736100) gene variations in parents and their newborns, revealing that the homozygous C/C genotype is prevalent in chronic diseases like diabetes and hypertension. In the TERC gene, the CC genotype was predominantly observed in newborns of parents with diabetes [16 (79%)] and COVID-19 [3 (60%)], though the association was not statistically significant (*p* = 0.33) (Supplementary Table 1). Similar genotype patterns have been reported in previous studies on related diseases [5, 28]. Moreover, newborns of parents with diabetes and hypertension showed significant associations with TERT gene variants (10 (50%),9 (56%) *p* = 0.01), supporting genetic inheritance patterns linking paternal and newborn TL, aligning with previous studies [29, 30]. However, a study found that TERT allele homozygotes had a lower prevalence of diabetes than heterozygotes (5.63% vs. 15.38%, *p* = 0.039) [31]. According to research on the genotype, the AC genotype was discovered to be a significant risk factor for "idiopathic pulmonary fibrosis" (IPF) in comparison to other lung diseases [29, 32, 33].

Telomere length, maintenance, and repair are influenced by genetic variations in telomerase genes, particularly polymorphisms associated with diseases. A study found that the genotype was associated with telomere shortening and disease, while showing maternal genotypes, more commonly inherited by newborns, consistent with the role of perinatal genetic and lifestyle factors [34–36]. Notably, in this study, the CC genotype in newborns may indicate a disease risk compared to parents with other genotypes.

The overarching effect of this study emphasized that parental health significantly influences newborn health and immune system development. Extensive literature exists on the impact of maternal risk factor modifications on newborn health [37, 38]. However, this study highlights for the first time that modifying fathers' external factors like social status and environment may have a progressive effect on both telomeres and the immune senescence of newborns. Different disease exposure is included in this study, which strengthens the results and adds data to the literature.

### Limitations

This study has several limitations. First, as a cross-sectional design, it involved a limited sample size, which may affect the strength of associations observed. Second, the absence of data on lifestyle, social determinants, and maternal nutrient deficiencies may have influenced immune senescence and telomere outcomes. Additionally, depending on self-reported information introduces potential bias. The analysis was also restricted to a limited set of immune senescence markers and telomerase gene polymorphism, which may not fully capture their role in disease development. Moreover, including mRNA expression and telomerase protein level monitoring in both parents and newborns could better elucidate the dynamics of telomere.

## Conclusions

It was found that parental clinical manifestation of diabetes is significantly associated with shorter telomeres, TERT gene polymorphism and up-regulation of the immune senescence markers, especially the KLRG1 in newborns. This is the first study to explore the associations between newborn TL, immune markers, and telomere maintenance genes (TERC and TERT) with parental telomere genetics, both globally and within a subset of Karachi, Pakistan.

## Supplementary Information

Supplementary material 1.

Supplementary material 2.

## Data Availability

The sequence data generated and analyzed during the current study are publicly available on the NCBI database under the following accession numbers: TERC: [OP046318–OP046360](https:/www.ncbi.nlm.nih.gov/nuccore/OP046318), TERT: [OP081479–OP081528](https:/www.ncbi.nlm.nih.gov/nuccore/OP081479) Data supporting telomere length measurements and flow cytometry analyses are not publicly available to protect patient privacy, but they may be obtained from the corresponding author upon reasonable request.
