# Supplementary material for: Parental clinical manifestation association with newborn immune senescence and telomere biology in Pakistan
Source: BMC Res Notes. 2025 Oct 30;18:461. doi: 10.1186/s13104-025-07498-4 (PMC12577043; doi:10.1186/s13104-025-07498-4)
Supplement: Supplementary file 1 — Supplementary material 1. [file 13104_2025_7498_MOESM1_ESM.pdf]

QUESTIONNAIRE

Identifier Number: ___________________ Phone No: _________________________
Female Age (yrs): ____________________Husband Age (yrs): __________________
Female Occupation: __________________ Husband occupation: ________________
Total Income: _______________________ Ethnicity: __________________________
Residence area: ____
Weight: __________________Height:____________BMI:_______________________
Gravida: ___________________ Parity: __________ No. of miscarriages: __________

Female Education:
No schooling: ☐
Matric: ☐
Inter: ☐
Graduation: ☐
Masters ☐
Madrassa: ☐

Husband Education:
No schooling: ☐
Matric: ☐
Inter: ☐
Graduation: ☐
Masters: ☐
Madrassa: ☐

Tobacco consumption (female):Smoker: ☐ Chewer: ☐
Tobacco consumption (Husband): Smoker: ☐ Chewer: ☐
Regular Medicine/Drugs (Female): _________Regular Medicine/Drugs (Husband): ____________
Blood group (Female): _______________ Blood group (Husband): ______________

Disease History (yes/No)

**Diseases**	**Female**	**Female Family**	**Husband**	**Husband Family**
Consanguinity marriage				
Diabetes				
Gestational Diabetes Mellitus				
Hypertension				
Preeclampsia				
Anemia				
COVID-19				
Others:				

**Newborn Characteristics**				
No. Of children	1st	2nd	3rd	4th
Mode of delivery	Normal ☐<br>C- section ☐	Normal ☐<br>C- section ☐	Normal ☐<br>C- section ☐	Normal ☐<br>C- section ☐
Gestational age				
Birth weight				
Gender				
Blood Group				
