# Supplementary material for: Parental clinical manifestation association with newborn immune senescence and telomere biology in Pakistan
Source: BMC Res Notes. 2025 Oct 30;18:461. doi: 10.1186/s13104-025-07498-4 (PMC12577043; doi:10.1186/s13104-025-07498-4)
Supplement: Supplementary file 2 — Supplementary material 2. [file 13104_2025_7498_MOESM2_ESM.pdf]

Supplementary Table 1: TERC genotype in parents and their newborns with chronic and acute diseases

Variables	TERC Genotype (n=53)									P value
	Mother n(%)			Father n(%)			Newborn n(%)			
	**CC 22(42)**	**TC 23(43)**	**TT 8(15)**	**CC 18(34)**	**TC 29(55)**	**TT 6(11)**	**CC 34(64)**	**TC 10(19)**	**TT 9(17)**	
**Chronic Diseases**										
Diabetes n=20	10 (50)	7 (36)	3 (14)	7 (36)	13 (64)	n/a	16(79)	1(7)	3 (14)	0.079
Hypertension n=16	4 (22)	7(44)	5(33)	6 (37)	8 (50)	2 (13)	8 (50)	4 (25)	4 (25)	0.78
Diabetes & Hypertension n=5	3 (60)	2 (40)	n/a	2 (40)	1(20)	2(40)	3 (60)	n/a	2 (40)	0.09
Anemia n=7	4(60)	3 (40)	n/a	3(40)	4(60)	n/a	4(60)	3 (40)	n/a	0.25
P-value	0.45			0.93			0.89			
**Acute Disease**										
COVID-19 n=5	1(20)	4 (80)	n/a	n/a	3(60)	2 (40)	3(60)	2 (40)	n/a	0.46
P-value	0.92			0.67			0.33			
n/a: not available *P value 0.05 significant										

Supplementary Table 2: TERT genotype in parents and their newborns with chronic and acute diseases

Variables	TERT Genotype (n=53)									P vlaue
	Mother n(%)			Father n(%)			Newborn n(%)			
	**CC 24 (46)**	**AC 29 (54)**	**AA n/a**	**CC 17 (32)**	**AC 27 (51)**	**AA 9 (17)**	**CC 28 (53)**	**AC 21 (40)**	**AA 4 (7)**	
**Chronic Diseases**										
Diabetes n=20	13 (64)	7 (36)	n/a	10 (50)	9(43)	1(7)	10 (50)	7 (35)	3 (15)	0.00*
Hypertension n=16	6 (40)	10 (60)	n/a	5 (32)	6 (36)	5 (32)	9 (56)	7 (44)	n/a	0.03*
Diabetees & Hypertension n=5	1 (20)	4 (80)	n/a	n/a	2 (40)	3 (60)	1 (20)	3 (60)	1 (20)	0.00*
Anemia n=7	1 (20)	6 (80)	n/a	1 (40)	6 (60)	n/a	5 (71)	2 (29)	n/a	0.89
P value	0.02			0.06			0.01			
**Acute Diseases**										
COVID n=5	3(60)	2(40)	n/a	1 (20)	4 (80)	n/a	3 (60)	2 (40)	n/a	0.045*
P value	0.34			0.54			0.01			
n/a: not available *P value 0.05 significant										

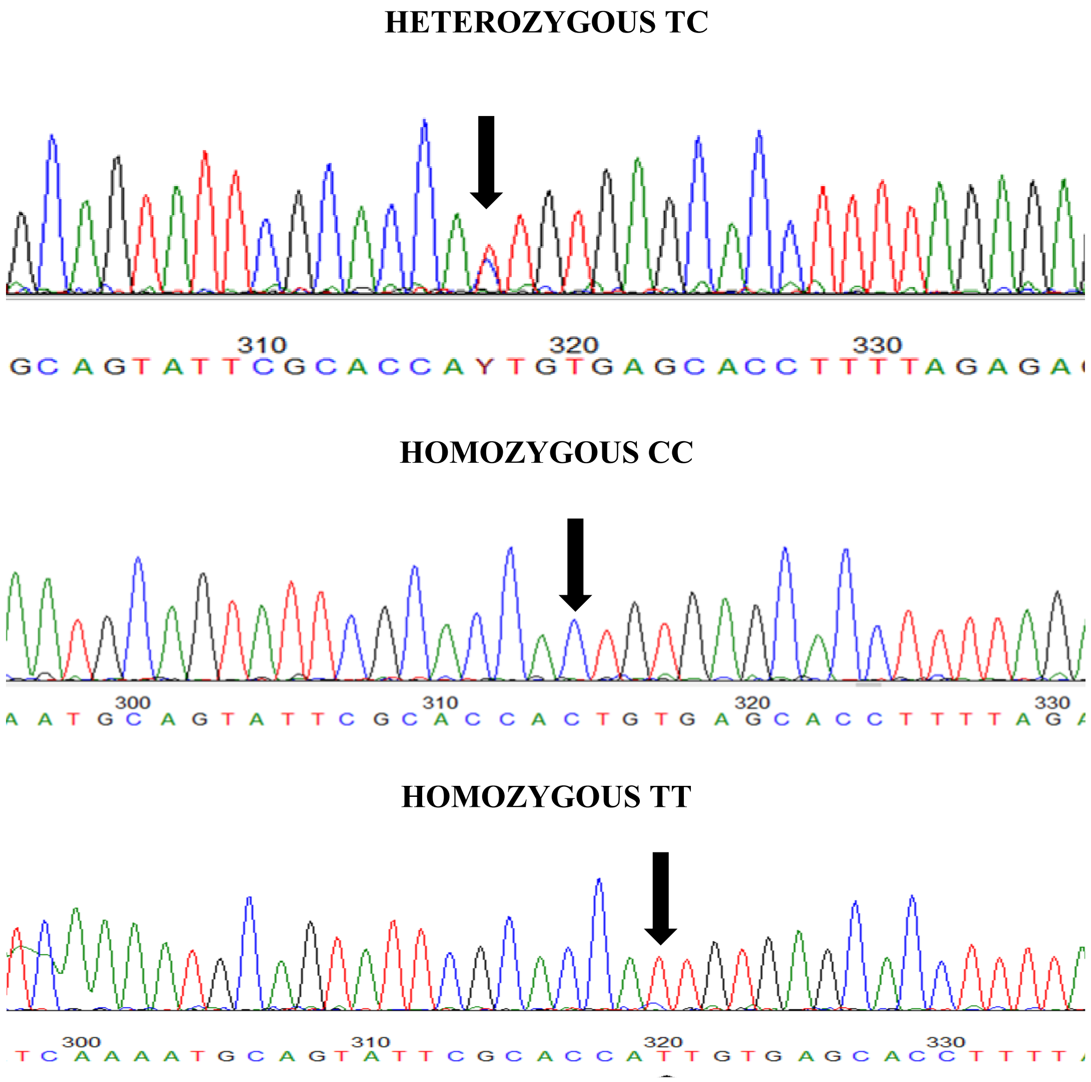

Supplementary Figure 1: Electropherogram of the TERC gene highlighting SNP (10936599)

HETEROZYGOUS AC

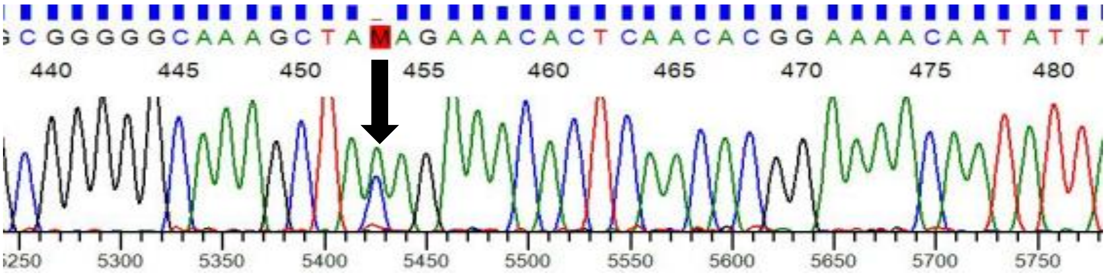

HOMOZYGOUS CC

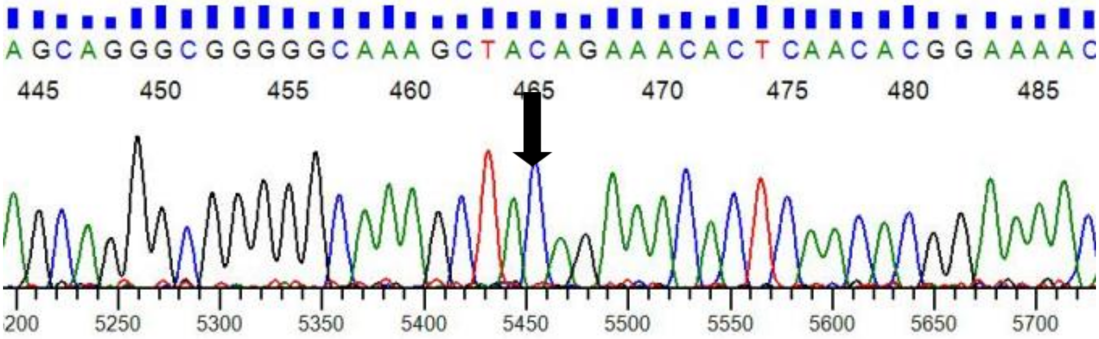

HOMOZYGOUS AA

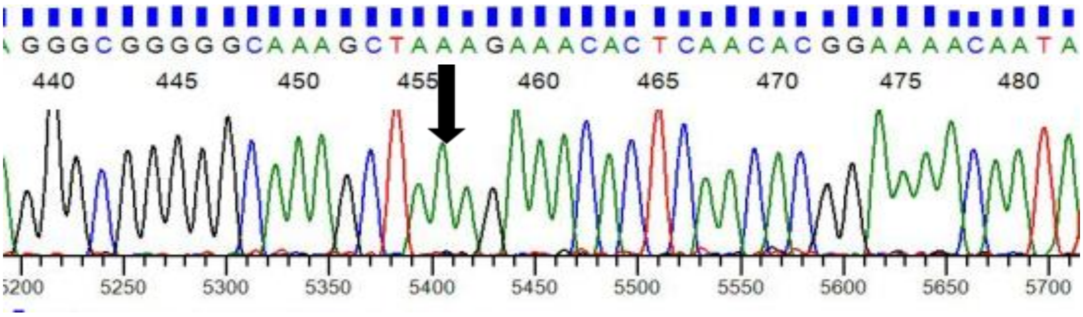

Supplementary Figure 2: Electropherogram of TERT gene highlighting SNP (rs2736100)

Supplementary Table 3: Correlation analysis among immune senescence markers CD57 and KLRG1 of diseased parents and their newborns

Variables		CD57-Mother	CD57 KLRG1-Mother	KLRGI-Mother	CD57-Father	CD57 KLRG1-Father	KLRGI-Father	CD57-Newborn	CD57 KLRG1-Newborn	KLRGI-Newborn
CD57-Mother	r	1.000	.610	.258	.206	.161	-.055	-.098	.293	.376
	P-value	.	.000	.058	.131	.241	.691	.474	.030	.005
CD57 KLRG1-Mother	r	.610	1.000	.496	.031	.172	.115	.028	.286	.525
	P-value	.000	.	.000	.820	.209	.402	.840	.035*	.000
KLRGI-Mother	r	.258	.496	1.000	.150	.192	.203	-.081	.119	.583
	P-value	.058	.000	.	.274	.159	.138	.556	.387	.000*
CD57-Father	r	.206	.031	.150	1.000	.669	-.005	.287	.332	.475
	P-value	.131	.820	.274	.	.000	.970	.034	.013	.000
CD57 KLRG1-Father	r	.161	.172	.192	.669	1.000	.449	.258	.288	.573
	P-value	.241	.209	.159	.000	.	.001	.057	.033*	.000*
KLRGI-Father	r	-.055	.115	.203	-.005	.449	1.000	.167	.012	.247
	P-value	.691	.402	.138	.970	.001	.	.222	.929	.069
CD57-Newborn	r	-.098	.028	-.081	.287	.258	.167	1.000	.385	-.032
	P-value	.474	.840	.556	.034	.057	.222	.	.004	.818
CD57 KLRG1-Newborn	r	.293	.286	.119	.332	.288	.012	.385	1.000	.456
	P-value	.030	.035	.387	.013	.033	.929	.004	.	.000
KLRGI-Newborn	r	.376	.525	.583	.475	.573	.247	-.032	.456	1.000
	P-value	.005	.000	.000	.000	.000	.069	.818	.000	.

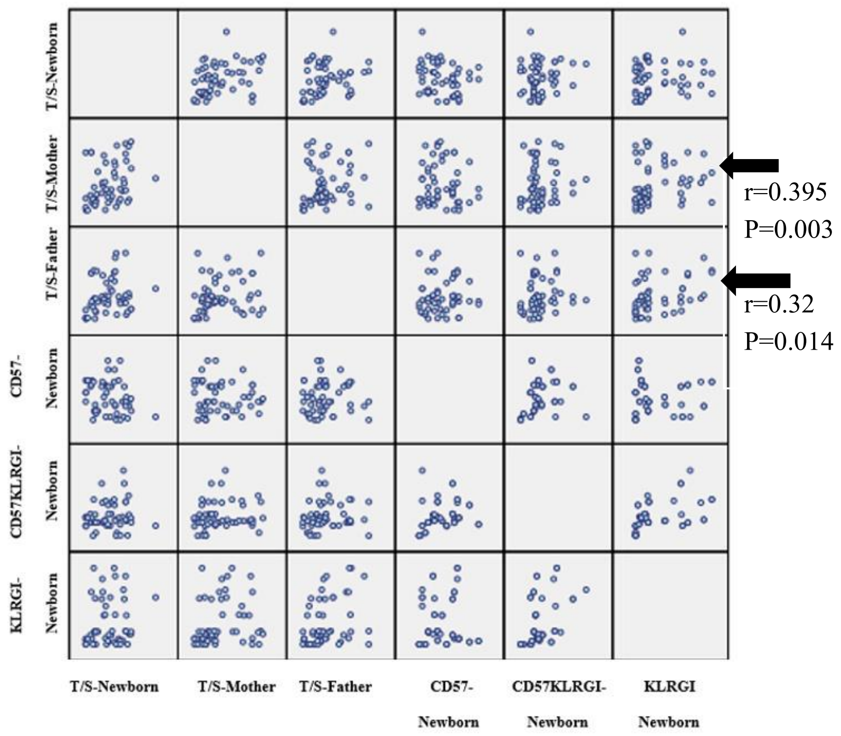

Supplementay Figure **3**: Correlation matrix with scatter plot between TL and immune senescence markers of parents and newborns.

.
